# Nutrition Facts Label Use and Adherence to the DASH Dietary Pattern: Results From a National Health and Nutrition Survey

**DOI:** 10.5888/pcd22.240426

**Published:** 2025-06-26

**Authors:** Miri Lutski, Aliza H. Stark, Rita Dichtiar, Shay Y. Lubel, Efrat Monsonego Ornan, Tali Sinai

**Affiliations:** 1 School of Nutritional Sciences, The Robert H. Smith Faculty of Agriculture, Food and Environment, The Hebrew University of Jerusalem, Rehovot, Israel; 2 Israel Center for Disease Control, Israel Ministry of Health, Ramat Gan, Israel; 3 School of Public Health, Faculty of Medical and Health Sciences, Tel Aviv University, Tel-Aviv, Israel

## Abstract

**Introduction:**

Whether using nutrition facts labels (NFLs) leads to adherence to higher-quality dietary patterns — such as the Dietary Approaches to Stop Hypertension (DASH) diet, which is associated with lower risk of chronic diseases — is unclear. We investigated whether people aged 21 to 64 years who use NFLs were more likely to adhere to the DASH dietary pattern.

**Methods:**

We analyzed data from the nationally representative, cross-sectional Israeli National Health and Nutrition Survey for Adults from 2014–2016. Adherence to the DASH diet was determined from a single 24-hour dietary recall using a DASH score calculated on the basis of adherence to 9 nutrient targets. Multivariable logistic regression was used to estimate odds ratios (ORs) and 95% CIs for DASH accordance (DASH score ≥4.5 [accordant] vs DASH score <4.5 [nonaccordant]) and for each separate nutrient target of the DASH diet.

**Results:**

The data set comprised 2,579 participants (NFL users, n = 931 [36.1%]). Of the NFL users, 299 (32.1%) were classified as DASH accordant, compared with 339 (20.6%) of the non-NFL users. After adjusting for potential confounders, the OR for DASH adherence among NFL users was 1.52 (95% CI, 1.20–1.93) compared with non-NFL users. Compared with non-NFL users, NFL users’ odds of meeting individual DASH nutrient targets were 1.30 (95% CI, 1.06–1.59) for protein; 1.46 (95% CI, 1.17–1.81) for dietary fiber; 1.48 (95% CI, 1.18–1.85) for magnesium; 1.38 (95% CI, 1.12–1.70) for calcium; and 1.60 (95% CI, 1.30–1.97) for potassium. Age, female sex, and performing recommended physical activity were associated with DASH adherence.

**Conclusion:**

These results highlight the importance of nutrition education and awareness, as well as the potential role of NFLs in promoting healthier dietary habits.

**Summary**

**What is known on this topic?**

Nutrition facts labels (NFLs) can promote healthier food choices by reducing energy intake and increasing vegetable consumption. However, the effect of NFLs on adherence to comprehensive dietary patterns, such as the Dietary Approaches to Stop Hypertension (DASH) diet, has not been thoroughly investigated.

**What is added by this report?**

This study demonstrated that people who regularly use NFLs are more likely to adhere to the DASH dietary pattern. Specifically, NFL users showed higher adherence to nutrient targets for protein, dietary fibers, magnesium, calcium, and potassium, compared with nonusers.

**What are the implications for public health practice?**

NFLs can support adherence to beneficial dietary patterns like the DASH diet. Policymakers and health educators could leverage these insights to improve public health strategies and design more effective food labeling systems.

## Introduction

Nutrition facts labels (NFLs) are standardized labels found on packaged foods in many countries that enable consumers to make informed decisions about food choices (1). These labels provide detailed information about the nutritional content of the food product, such as energy (ie, calories), macronutrients (ie, fats, carbohydrates, and proteins), micronutrients (eg, vitamins, minerals), and other relevant nutrients such as dietary fibers and sugar. NFLs specify the serving size for which the nutritional information is provided and allow easy comparison among products (1).

In many places, including the US, Canada, and Europe, NFLs are mandatory on most packaged foods. These labels are regulated by government agencies such as the US Food and Drug Administration (2) or the European Food Safety Authority in the European Union. In Israel, these practices are compulsory and regulated by law, primarily through the Consumer Protection Order (Marking and Packaging of Food Products) of 1998 (3). This law outlines strict rules for food labeling in Israel and requires detailed ingredient lists; nutritional information such as calories, fats, carbohydrates, and proteins per 100 g; allergen warnings; expiration dates; and contact details of manufacturers to ensure consumer safety and awareness. Systematic reviews and meta-analyses have shown that using NFLs is linked with adopting healthier dietary habits and favoring healthier products, as well as higher vegetable intake and reduced consumption of energy and total fat (4–6). Despite these advantages, NFLs do not provide context about overall dietary patterns or the role of specific foods within a balanced diet (7). Additionally, NFLs can be complex and overwhelming for some consumers to understand. They may struggle to interpret the information provided, especially regarding serving sizes and nutrient measurements (7,8). Thus, whether the use of NFLs leads to adherence to a higher quality diet pattern beyond its effect on the reduction in energy consumption and the intake of a specific component such as fats, sugar, or salt is unclear.

The Dietary Approaches to Stop Hypertension (DASH) diet is a widely recognized dietary pattern designed to reduce blood pressure (9). It emphasizes the consumption of fruits, vegetables, whole grains, lean proteins, and low-fat dairy products while limiting the intake of saturated fats, cholesterol, and sodium (9). Moreover, the DASH diet is recognized for its heart-healthy benefits, including improvements in lipid profile (10), promotion of weight loss (11), mitigation of the risk of type 2 diabetes (12), prevention of cognitive decline (13), and reduction in all-cause mortality (14). However, the correlation between NFL use and adherence to dietary patterns like the DASH diet, which can signify commitment to a healthy lifestyle, has not been investigated. Therefore, we aimed to determine whether adults who use NFLs were more likely to adhere to the DASH dietary pattern.

## Methods

### Study design

This cross-sectional study was based on data obtained from the second representative cross-sectional Israeli National Health and Nutrition Survey, known as Rav Mabat Adult (15). Specifically, this survey assessed Israeli community-dwelling adults aged 18 to 64 years and was conducted during 2014–2016 by the Israel Center for Disease Control (ICDC), the Nutrition Division of the Israel Ministry of Health, and the Israel Central Bureau of Statistics (CBS) (15). Detailed descriptions of the survey design, questionnaires, and procedures can be found on the ICDC website (15). Briefly, a national random stratum sampling was performed, according to population group (Jewish adults/Arab adults) and locality. Additionally, the data were weighted for the total population by weighting factors created by the CBS and were based on age, population group, sex, education, place of birth, and dietary recall day. Trained personnel performed face-to-face interviews, focusing on demographic characteristics, health status, lifestyle, dietary characteristics, and anthropometric measurements.

The survey received ethical approval from the Israeli Ministry of Health, and all participants provided written informed consent.

### Study population

Of the 2,957 adults aged 18 to 64 years, 132 participants (4.5%) aged 18 to 20 years were excluded to focus on adults who were not in mandatory military service. Among the remaining 2,825 participants aged 21 to 64 years, 246 (8.7%) were excluded due to missing NFL use data, missing dietary data, unusual diary food evaluations resulting from illness or fasting, or invalid dietary recalls — defined as total energy intake of less than 500 kcal/d or more than 5,000 kcal/d. The final data set included 2,579 participants.

### Evaluation of nutrition facts labels use

The use of NFLs was defined by the following question: “When you read the information on the food label, do you check the nutrition facts, which lists calories, carbohydrates, etc, per 100 g of food?” Participants who answered “always or often” were categorized as NFLs users, while participants who answered “rarely or never” were categorized as non-NFLs users.

### The DASH diet assessment

A structured face-to-face interview with a focus on food and beverage intake in the preceding 24 hours (single-day, 24-hour recall) was used (16). To enhance accuracy, respondents were assisted with measuring aids, pictures, and other visual tools from *The Israeli Food and Food Quantities Guide* to describe food and portion sizes (15). Energy (kcal/d) and macronutrient (g/d) intakes were calculated using the Tzameret software program based on the Israeli food and nutrient database (17).

The DASH score was determined on the basis of adherence to 9 target nutrients, as established in previous work by Mellen et al (18) and used by others (19,20–22): saturated fatty acids (SFA) (≤6% of energy), total fat (≤27% of energy), protein (≥18% of energy), cholesterol (≤71.4 mg/1,000 kcal), dietary fiber (≥14.8 g/1,000 kcal), magnesium (≥238 mg/1,000 kcal), calcium (≥590 mg/1,000 kcal), potassium (≥2,238 mg/1,000 kcal), and sodium (≤1,143 mg/1,000 kcal). Participants were awarded 1 point for meeting the goal for each nutrient, and 0.5 points if they achieved the intermediate goal, with a maximum score of 9. Adults with a DASH score of 4.5 points or more were classified as DASH accordant, consistent with prior studies (18–22). This threshold has been used to differentiate adherence levels in studies evaluating the relationship between DASH adherence and health outcomes.

### Additional assessments

Demographic and health characteristic data were collected systematically through face-to-face interviews conducted in the respondents’ homes by trained interviewers. Educational level was divided into 2 categories: elementary and high school (0–12 y) and any postsecondary education (more than 12 years of schooling). Socioeconomic status (SES) was defined according to the National Insurance Institute, based on income and family size and defined as above or below the poverty line. Population group was defined based on the definitions used by the Israel CBS as Jews and Arabs. Smoking status was classified as ever (current and past) or never. Body mass index (BMI) was calculated as weight in kilograms divided by the square of height in meters. Blood pressure status and hypercholesterolemia were determined through self-report, based on physician’s diagnosis. Participants were classified as meeting the World Health Organization recommendations for adults aged 18 to 64 years if they engaged in at least 150 minutes of moderate-intensity aerobic physical activity per week, or 75 minutes of vigorous-intensity aerobic physical activity per week, or an equivalent combination of moderate- and vigorous-intensity activities per week (23). Comorbidity, measured as the number of diseases per person, relied on self-reports of chronic illnesses diagnosed by a physician. These included type 2 diabetes, coronary heart disease (CHD), stroke, fatty liver, thyroid disease, asthma, anemia, osteoporosis, malignancy, and Parkinson disease. Participants were categorized as having low comorbidity if they reported 0 or 1 of these diseases and high comorbidity if they reported 2 or more diseases.

### Statistical analysis

The one-sample Kolmogorov–Smirnov test was applied to test for a normal distribution of continuous variables. The clinical characteristics of the participants were expressed as frequencies and percentages for categorical variables and as median and IQR for continuous variables, due to the strongly skewed distribution. Chi-square and Mann–Whitney tests were performed to compare the demographic, health, and diet characteristics of subject groups and NFL use, and by DASH-accordant groups. Multivariable logistic regression was used to estimate odds ratios (ORs) and 95% CIs for DASH accordance (DASH score ≥4.5 vs <4.5) and for each separate nutrient target of the DASH score. Model 1 adjusted for age, sex (women vs men), education (>12 y vs ≤12 y), population group (Arabs vs Jews), and marital status (married vs unmarried). Model 2 additionally adjusted for BMI, smoking (ever vs never), physical activity as recommended (yes vs no), hypertension (yes vs no), hypercholesterolemia (yes vs no), and comorbidities (high ≥2 vs low <2 chronic diseases). Potential interactions were assessed between all covariates and NFL use. Sensitivity analysis was conducted using multivariable logistic regression on a subset of participants with complete SES data (n = 2,011) due to the low response rate for SES (74.2%). Statistical analysis was performed using SAS Enterprise Guide version 7.12 (SAS Institute, Inc). Significance was set at *P* < .05.

## Results

Compared with 1,648 non-NFL users, NFL users (n = 931) were older and more educated, with higher proportions of women, Jews, and nonsmokers (Table 1). NFL users exhibited significantly higher levels of physical activity, prevalence of hypercholesterolemia, prevalence of comorbidities (≥2 chronic diseases), and proportion of adults living above the poverty line. The groups showed similarities in BMI distribution and the prevalence of chronic diseases such as type 2 diabetes, CHD, stroke, thyroid disease, asthma, anemia, osteoporosis, malignancy, and Parkinson disease. Demographic and health characteristics based on DASH accordance can be found at https://doi.org/10.6084/m9.figshare.29145452.v4. A significantly higher prevalence of DASH accordance was found among women and among people with hypertension, high BMI, thyroid disease, diabetes, and comorbidities.

**Table 1:** Demographic and Health Characteristics of Adults,^a^ by Use of Nutrition Facts Labels (NFLs), Israeli National Health and Nutrition Survey for Adults, 2014–2016

Characteristic	No.	Total (N = 2,579)	NFL users, n = 931 (36.1%)	Non-NFL users, n = 1,648 (63.9%)	*P* value
**Demographic**					
Age, median (IQR), y^b^	2,579	40 (31–49)	41 (32–51)	39 (31–48)	.007
**Sex, no. (%)**					
Male	2,579	1,226 (49.7)	371 (41.5)	855 (54.2)	<.001
Female		1,353 (50.3)	560 (58.5)	793 (45.8)	
**Education, no. (%), y**					
0–12	2,556	1,064 (44.4)	291 (33.7)	773 (50.5)	<.001
≥13		1,492 (55.6)	633 (66.3)	859 (49.5)	
**Marital status, no. (%)**					
Unmarried	2,575	736 (34.6)	279 (35.3)	457 (34.2)	.23
Married		1,839 (65.4)	651 (64.7)	1,188 (65.8)	
**Socioeconomic status, no. (%)**					
Above poverty line	2,579	1,588 (58.4)	615 (66.1)	973 (59.0)	<.001
Below poverty line		423 (15.7)	112 (12.0)	311 (18.9)	
Missing		568 (25.8)	204 (21.9)	364 (22.1)	
**Population group, no. (%)**					
Jewish adults	2,579	2,120 (80.7)	826 (86.8)	1,294 (77.2)	<.001
Arab adults		459 (19.3)	105 (13.2)	354 (22.8)	
**Health and lifestyle**					
BMI, median (IQR), kg/m^2b^	1,871	25 (22–28)	25 (22–28)	25 (22–29)	.38
**Smoking status, no. (%)**					
Ever	2,573	732 (28.6)	206 (22.0)	526 (32.4)	<.001
Never		1,841 (71.4)	724 (78.0)	1,117 (67.6)	
Physical activity as recommended, no. (%)	2,538	839 (33.0)	411 (45.5)	428 (26.0)	<.001
Hypertension, no. (%)	2,560	395 (16.2)	156 (17.5)	239 (15.4)	.12
Hypercholesterolemia, no. (%)	2,550	277 (11.1)	127 (14.0)	150 (9.5)	<.001
**Nutritional intake**					
Energy, median (IQR), kcal/d^b^	2,579	1,524 (1,124–2,052)	1,406 (1,070–1,898)	1,605 (1,179–2,152)	<.001
DASH score, median (IQR)^b^^,^ c	2,579	3.0 (2.0–4.0)	3.5 (2.5–4.5)	3.0 (2.0–4.0)	<.001
**Morbidity**					
Diabetes, no. (%)	2,576	213 (8.6)	86 (9.5)	127 (8.1)	.17
Coronary heart disease, no. (%)	2,569	98 (4.1)	30 (3.3)	68 (4.6)	.25
Stroke, no. (%)	2,571	43 (1.5)	14 (1.3)	29 (1.7)	.63
Fatty liver, no. (%)	2,568	145 (5.5)	72 (7.6)	73 (4.4)	<.001
Thyroid disease, no. (%)	2,569	205 (7.8)	86 (9.1)	119 (7.1)	.07
Asthma, no. (%)	2,571	196 (7.6)	75 (8.6)	121 (7.0)	.52
Anemia, no. (%)	2,568	651 (23.2)	251 (25.2)	400 (22.2)	.13
Osteoporosis, no. (%)	2,563	110 (4.4)	49 (5.4)	61 (3.9)	.06
Malignancy, no. (%)	2,572	79 (2.9)	33 (3.4)	46 (2.5)	.29
Parkinson disease, no. (%)	2,569	32 (1.0)	10 (0.8)	22 (1.1)	.56
**Comorbidity, no. (%)**					
High (≥2 chronic diseases)	2,548	326 (12.4)	138 (14.7)	188 (12.5)	.01
Low (<2 chronic diseases)		2,222 (87.6)	783 (85.3)	1,439 (87.5)	

Abbreviations: BMI, body mass index; DASH, Dietary Approaches to Stop Hypertension.

a All data were weighted.

b Median and IQR are presented for variables with skewed distributions.

c The DASH score was determined based on adherence to 9 target nutrients, as established by Mellen et al (18): saturated fatty acids, ≤6% of energy; total fat, ≤27% of energy; protein, ≥18% of energy; cholesterol, ≤71.4 mg/1,000 kcal; dietary fiber, ≥14.8 g/1,000 kcal; magnesium, ≥238 mg/1,000 kcal; calcium, ≥590 mg/1,000 kcal; potassium, ≥2,238 mg/1,000 kcal; and sodium, ≤1,143 mg/1,000 kcal. Participants were awarded 1 point for meeting the goal for each nutrient, and 0.5 points if they achieved the intermediate goal, with a maximum score of 9. Adults with a DASH score of 4.5 points or more were classified as DASH accordant.

**Figure 1:**
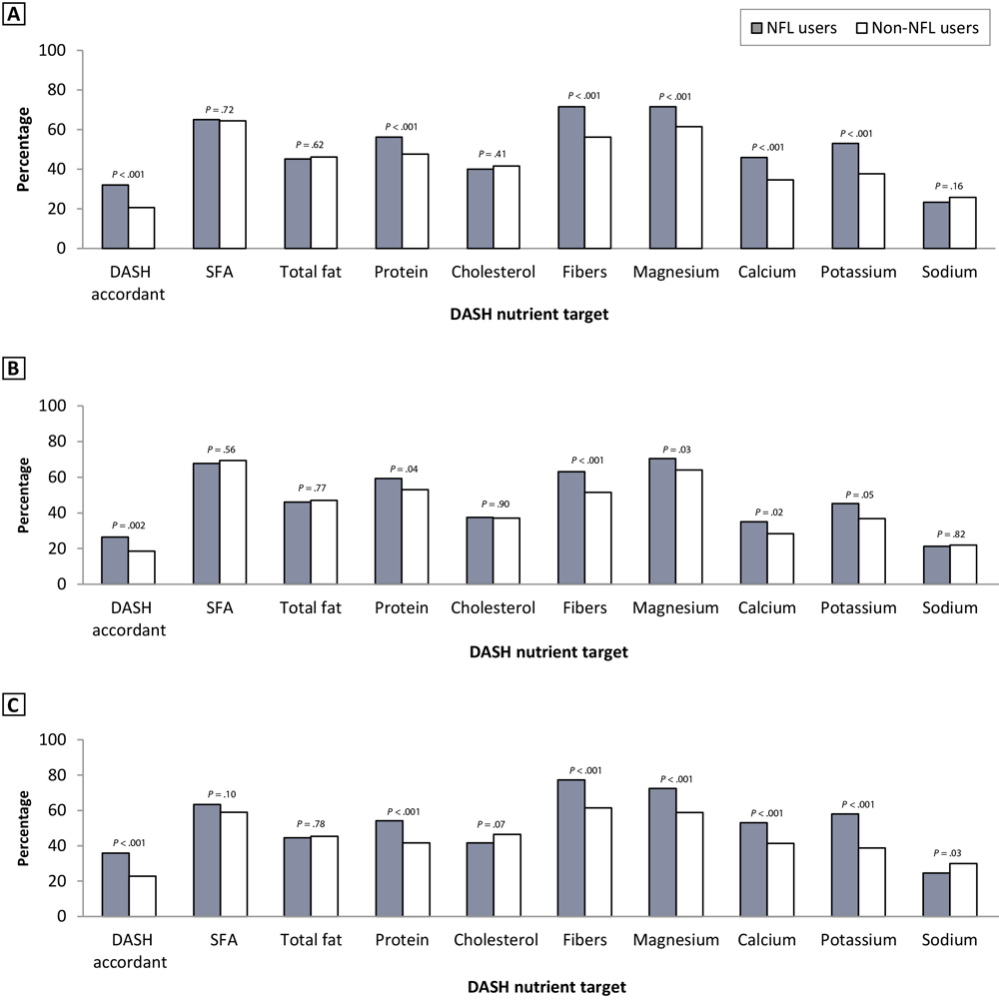
Distribution of DASH accordance and DASH nutrient targets, by NFL user groups overall and by sex (A, all users; B, male users; C, female users). DASH score was determined based on adherence to 9 target nutrients, as established by Mellen et al (18). DASH nutrient score targets: DASH accordant, score ≥4.5; SFA, ≤6% of energy; total fat, ≤27% of energy; protein, ≥18% of energy; cholesterol, ≤71.4 mg/1,000 kcal; dietary fibers, ≥14.8 g/1,000 kcal; magnesium, ≥238 mg/1,000 kcal; calcium, ≥590 mg/1,000 kcal; potassium, ≥2,238 mg/1,000 kcal; sodium, ≤1,143 mg/1,000 kcal. Abbreviations: DASH, Dietary Approaches to Stop Hypertension; NFL, Nutrition Facts Label; SFA, saturated fatty acids.

Figure 1 illustrates the rates of people who were classified as DASH adherent, by NFL use and by sex. Overall, 299 (32.1%) of the NFL users were classified as DASH accordant, compared with 339 (20.6%) of non-NFL users. NFL users also had higher rates of DASH adherence and better achievement of nutrient targets such as protein (56.2% vs 47.6%), dietary fiber (71.6% vs 56.2%), magnesium (71.6% vs 61.5%), calcium (45.9% vs 34.6%), and potassium (53.0% vs 37.7%). No significant differences were found for SFA, total fat, cholesterol, and sodium. Compared with male NFL users, female NFL users also showed higher accordance with potassium and sodium DASH nutrient targets (Figure 1).

**Table:** 

DASH nutrient target	All, %	*P* value	Male users, %	*P* value	Female users, %	*P* value
**DASH accordant**						
NFL users	32.1	<.001	26.4	.002	35.9	<.001
Non-NFL users	20.6		18.5		22.8	
**Saturated fatty acids**						
NFL users	65.1	.72	67.7	.56	63.4	.10
Non-NFL users	64.4		69.4		59.0	
**Total fat**						
NFL users	45.2	.62	46.1	.77	44.6	.78
Non-NFL users	46.2		47.0		45.4	
**Protein**						
NFL users	56.2	<.001	59.3	.04	54.1	<.001
Non-NFL users	47.6		53.0		41.7	
**Cholesterol**						
NFL users	40.0	.41	37.5	.90	41.6	.07
Non-NFL users	41.6		37.1		46.5	
**Fibers**						
NFL users	71.6	<.001	63.1	<.001	77.3	<.001
Non-NFL users	56.2		51.5		61.4	
**Magnesium**						
NFL users	71.6	<.001	70.4	.03	72.5	<.001
Non-NFL users	61.5		64.0		58.8	
**Calcium**						
NFL users	45.9	<.001	35.0	.02	53.0	<.001
Non-NFL users	34.6		28.3		41.4	
**Potassium**						
NFL users	53.0	<.001	45.3	.05	58.0	<.001
Non-NFL users	37.7		36.8		38.7	
**Sodium**						
NFL users	23.3	.16	21.3	.82	24.6	.03
Non-NFL users	25.8		21.9		30.0	

After adjusting for age, sex, education, population group, and marital status (model 1), the estimated OR for DASH accordance among participants in the NFL user group was 1.72 (95% CI, 1.42–2.09) compared with non-NFL users (Table 2). In further adjustments for health-related behaviors, BMI, and morbidities (model 2), the estimated OR for DASH-accordance among NFL users was 1.52 (95% CI, 1.20–1.93) compared with non-NFL users. Compared with non-NFL users, NFL users’ odds of meeting individual DASH nutrient targets were 1.30 (95% CI, 1.06–1.59) for protein; 1.46 (95% CI, 1.17–1.81) for dietary fiber; 1.48 (95% CI, 1.18–1.85) for magnesium; 1.38 (95% CI, 1.12–1.70) for calcium; and 1.60 (95% CI, 1.30–1.97) for potassium.

**Table 2:** Multivariable Logistic Regression Analysis of Adults Who Were DASH Accordant^a^ and Met DASH Nutrient Targets, by Use of Nutrition Facts Labels (NFLs), Israeli National Health and Nutrition Survey for Adults, 2014–2016

Outcome	Model 1^b^		Model 2^b^	
	OR (95% CI)	*P* value	OR (95% CI)	*P* value
**DASH accordant (DASH score ≥4.5)**				
NFL users	1.72 (1.42–2.09)	<.001	1.52 (1.20–1.93)	.001
Non-NFL users	1 [Reference]		1 [Reference]	
**Saturated fatty acids (≤6% of energy)**				
NFL users	1.15 (0.96–1.36)	.13	1.04 (0.84–1.29)	.69
Non-NFL users	1 [Reference]		1 [Reference]	
**Total fat (≤27% of energy)**				
NFL users	1.02 (0.86–1.20)	.85	0.95 (0.78–1.17)	.64
Non-NFL users	1 [Reference]		1 [Reference]	
**Protein (≥18% of energy)**				
NFL users	1.42 (1.20–1.68)	<.001	1.30 (1.06–1.59)	.01
Non-NFL users	1 [Reference]		1 [Reference]	
**Cholesterol (≤71.4 mg/1,000 kcal)**				
NFL users	0.94 (0.80–1.12)	.49	0.96 (0.78–1.18)	.67
Non-NFL users	1 [Reference]		1 [Reference]	
**Dietary fibers (≥14.8 g/1,000 kcal)**				
NFL users	1.73 (1.44–2.07)	<.001	1.46 (1.17–1.81)	.001
Non-NFL users	1 [Reference]		1 [Reference]	
**Magnesium (≥238 mg/1,000 kcal)**				
NFL users	1.51 (1.26–1.82)	<.001	1.48 (1.18–1.85)	.001
Non-NFL users	1 [Reference]		1 [Reference]	
**Calcium (≥590 mg/1,000 kcal)**				
NFL users	1.46 (1.23–1.74)	<.001	1.38 (1.12–1.70)	.003
Non-NFL users	1 [Reference]		1 [Reference]	
**Potassium (≥2,238 mg/1,000 kcal)**				
NFL users	1.65 (1.39–1.96)	<.001	1.60 (1.30–1.97)	.007
Non-NFL users	1 [Reference]		1 [Reference]	
**Sodium (≤1,143 mg/1,000 kcal)**				
NFL users	0.83 (0.69–1.01)	.07	0.90 (0.71–1.14)	.38
Non-NFL users	1 [Reference]		1 [Reference]	

Abbreviations: BMI, body mass index; DASH, Dietary Approaches to Stop Hypertension; OR, odds ratio.

a The DASH score was determined based on adherence to 9 target nutrients, as established by Mellen et al (18): saturated fatty acids, ≤6% of energy; total fat, ≤27% of energy; protein, ≥18% of energy; cholesterol, ≤71.4 mg/1,000 kcal; dietary fiber, ≥14.8 g/1,000 kcal; magnesium, ≥238 mg/1,000 kcal; calcium, ≥590 mg/1,000 kcal; potassium, ≥2,238 mg/1,000 kcal; and sodium, ≤1,143 mg/1,000 kcal. Participants were awarded 1 point for meeting the goal for each nutrient, and 0.5 points if they achieved the intermediate goal, with a maximum score of 9. Adults with a DASH score of 4.5 points or more were classified as DASH accordant.

b Model 1 was adjusted for age (continuous), sex (women vs men), population group (Arabs vs Jews), education (>12 vs ≤12 years), and marital status (married vs unmarried). Model 2 was additionally adjusted for BMI (continuous), smoking (ever vs never), physical activity as recommended (yes vs no), hypertension (yes vs no), hypercholesterolemia (yes vs no), and comorbidity (high ≥2 vs low <2 chronic diseases).

**Figure 2:**
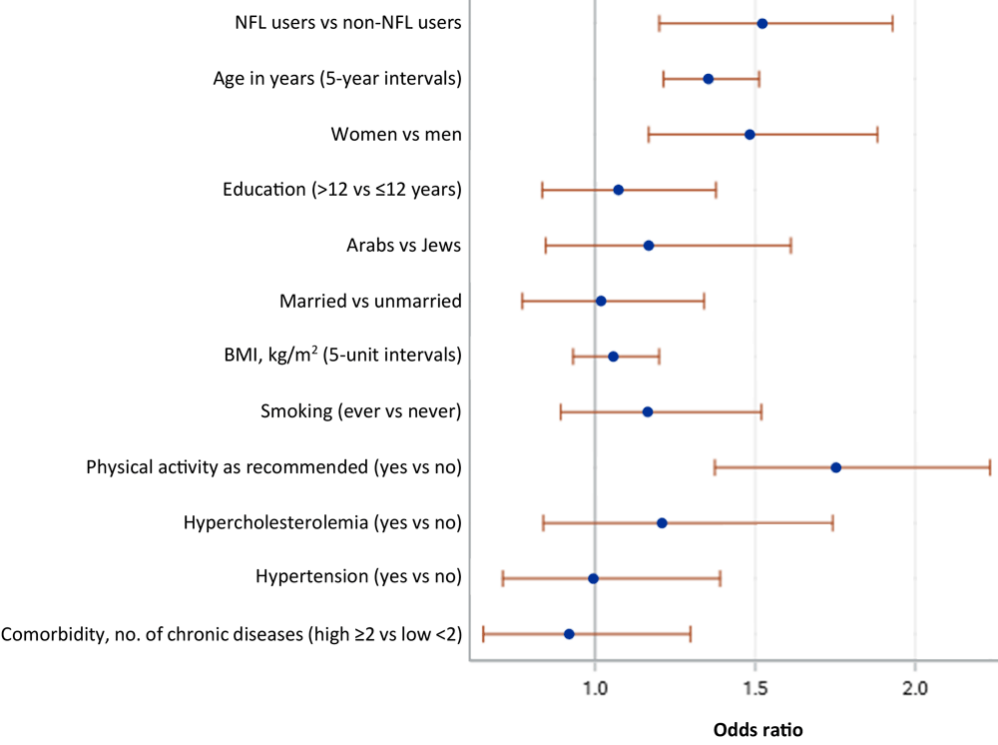
Odds of being accordant with the DASH diet (DASH score ≥4.5 vs <4.5), according to NFL user groups and covariates. DASH score was determined based on adherence to 9 target nutrients, as established by Mellen et al (18). Dots represent adjusted odds ratios and lines represent 95% confidence intervals. Abbreviations: BMI, body mass index; DASH, Dietary Approaches to Stop Hypertension; NFL, Nutrition Facts Label.

No significant differences were found for SFA, total fat, cholesterol, and sodium target intakes between NFL users versus non-NFL users (Table 2). Age, female sex, and physical activity as recommended were associated with DASH accordance (Figure 2). Similar point estimates were obtained when analyses were adjusted for SES (data can be accessed at https://doi.org/10.6084/m9.figshare.29145452.v4).

**Table:** 

Covariate	Odds ratio (95% confidence interval)
NFL users vs non-NFL users	1.52 (1.20–1.93)
Age in years (5-year intervals)	1.35 (1.21–1.51)
Women vs men	1.48 (1.17–1.88)
Education in years (>12 vs ≤12)	1.07 (0.84–1.38)
Arabs vs Jews	1.17 (0.85–1.61)
Married vs unmarried	1.02 (0.77–1.34)
BMI, kg/m^2^ (5-unit intervals)	1.06 (0.93–1.20)
Smoking (ever vs never)	1.16 (0.89–1.52)
Physical activity as recommended (yes vs no)	1.75 (1.37–2.23)
Hypercholesterolemia (yes vs no)	1.21 (0.84–1.74)
Hypertension (yes vs no)	0.99 (0.71–1.39)
Comorbidity, no. of chronic diseases (high ≥2 vs low <2)	0.92 (0.65–1.30)

Significant interaction was found only between NFL users, sex, and dietary fiber DASH nutrient target (*P* for interaction = .04). In subgroup analyses (Table 3), female NFL users were significantly associated with meeting the dietary fiber DASH nutrient target, with an adjusted OR of 2.13 (95% CI, 1.28–3.52); among male NFL users, the adjusted OR of meeting the dietary fiber nutrient target was 1.20 (95% CI, 0.77–1.87).

**Table 3:** Multivariable Logistic Regression of Adults Meeting DASH Target Intake of Dietary Fiber, by Use of Nutrition Facts Labels (NFLs) and by Sex, Israeli National Health and Nutrition Survey for Adults, 2014–2016^a^

Outcome	Men		Women		*P* value for interaction
	OR (95% CI)	*P* value	OR (95% CI)	*P* value	
**Fiber (≥14.8 g/1,000 kcal)**					
NFL users	1.20 (0.77–1.87)	.43	2.13 (1.28–3.52)	.003	.04
Non-NFL users	1 [Reference]		1 [Reference]		

Abbreviation: OR, odds ratio.

a Model was adjusted for age (continuous), sex (women vs men) population group (Jews vs Arabs), marital status (married vs unmarried), education (>12 y vs ≤12 y), body mass index (continuous), smoking (ever vs never), physical activity as recommended (yes vs no), hypertension (yes vs no), hypercholesterolemia (yes vs no), and comorbidity (high ≥2 vs low <2 chronic diseases).

## Discussion

After investigating the relationship between the use of NFLs and adherence to the DASH diet in adults aged 21 to 64 years, we found that adults who used NFLs may be more likely to adhere to the DASH dietary guidelines. Our findings indicate that NFL users had higher rates of achieving nutrient targets such as protein, dietary fiber, magnesium, calcium, and potassium. However, we found no significant differences for SFA, total fat, cholesterol, and sodium.

The overall prevalence of NFL use in Israeli adults was 36.1%. Reported prevalence of nutrition label use has varied substantially across studies. The prevalence rate of NFL use in our study was higher than NFL use in a study conducted in the United Kingdom (UK) (32%) (8). However, it was lower than those reported in studies conducted in the US (47%), Canada (47%), and Australia (43%), assessing the nationally representative population in each country (8). In a review of 16 studies based on data from college surveys in 4 countries (US, UK, Canada, and South Korea), the weighted prevalence of NFL use was 36.5% of college students and young adults aged 18 to 30 years (24). In the current study, 34.4% of participants aged 21 to 30 years reported using NFL (n = 593), a prevalence similar to the findings of this review. According to the findings of another telephone survey conducted in 2019 by a polling company, 59.3% of 513 Israeli adults aged 21 years or older reported that the information on food labels always or often influenced their decision to purchase a particular food product, while 22.8% said that the information never or rarely affects their decision (25).

Multiple studies have investigated the effect of food labeling on consumer behavior. A recent meta-analysis of 60 studies, encompassing 2 million observations from 111 intervention trials in 11 countries, found that food labeling led to a 6.6% reduction in consumer energy intake, a 10.6% reduction in total fat intake, and a 13.5% increase in vegetable consumption (6). Additionally, food labeling resulted in an 8.9% reduction in sodium content and a 64.3% decrease in artificial trans fat content in products (6). However, people do not consume isolated nutrients; they consume meals comprising complex combinations of multiple nutrients, and they have unique eating patterns (26).

To the best of our knowledge, only a few studies have examined the effect of using NFLs on adherence to complex dietary patterns (27), effective for health promotion and disease prevention (28–30). An Italian population-based cross-sectional study, which included a subsample of participants aged 35 years or older from the Moli-sani Project, found that use of food labels was associated with better adherence to the Mediterranean diet (27). Our study demonstrated that NFL users were more likely to adhere to the DASH diet, with 32.1% of NFL users classified as DASH adherent compared with 20.6% of nonusers. Female NFL users were significantly more likely to meet dietary fiber targets than male NFL users, with 77.3% of females achieving the target compared with 63.1% of males. These findings, along with those from the Italian study (27), provide evidence that food labels are linked not only to individual healthy food choices, as noted by meta-analysis (6), but also to an overall, healthier complex dietary pattern.

This study indicated that NFL users tend to be older and include a higher proportion of women compared with other studies (24,31). Additionally, our study demonstrated significantly higher NFL use by more physically active people. The trend suggested that NFL use may be more prevalent among populations with higher health awareness. The higher prevalence of physical activity among NFL users may reflect more engagement with health management and preventive measures (24). After adjustment for other variables, hypertension, hypercholesterolemia, and comorbidity status were not significantly associated with adherence to the DASH diet. Indeed, adherence to the DASH diet among people with these conditions has yielded mixed results across various studies. A recent cross-sectional study found no significant differences in hypertension status by DASH diet–adherent groups among Mexican men and women (32). Similarly, data from the National Health and Nutrition Examination Survey from 2007 to 2012 reported that people with hypertension exhibited lower adherence to DASH guidelines compared with those without hypertension (33). However, the Brazilian Longitudinal Study of Adult Health found that high adherence to the DASH diet was associated with a significant 26% lower risk of hypertension (hazard ratio = 0.74; 95% CI, 0.57–0.95) (34). This association, however, diminished to borderline significance after adjusting for BMI. These findings highlight the need for public health policies that promote adherence to healthy dietary patterns for the prevention of hypertension and its complications.

Our findings have several health policy implications. First, understanding and using NFL information effectively can help consumers make informed decisions about their food choices, manage their dietary intake, and maintain a balanced diet. It allows consumers to assess the nutritional content of various food products and compare them with recommended nutrient intakes. Second, the demographic characteristics associated with NFL usage suggest specific strategies to expand NFL adoption among diverse populations, particularly those from lower socioeconomic backgrounds or minority groups. Third, this research highlights the importance of nutrition education and awareness, as well as the role of tools like NFLs in promoting healthier dietary habits, including the DASH diet to manage hypertension. However, NFLs can be overwhelming and difficult to understand for some consumers, particularly those with limited nutritional knowledge and low levels of education. Research suggests that front-of-package labels (FOPLs) can influence purchasing decisions more effectively by providing clear guidance on healthier choices (35,36). FOPLs are more likely to catch consumers’ attention compared with NFLs on the back or side of the package (35,36). However, FOPLs may not offer the comprehensive detail found on NFLs and can lead to misinterpretation, especially if consumers do not understand the criteria behind the ratings or symbols (35,36). FOPLs and NFLs can complement each other. FOPLs may help provide quick, easy-to-understand information to guide initial decisions, while NFLs offer detailed data for those who want to delve deeper (37).

Strengths of this study include the use of a unique, nationally representative data set of extensively studied participants. The quality of the data collected is high since it adheres to stringent protocols and provides comprehensive training for the interviewers. However, our study has limitations, the main one being the cross-sectional design, which limits inferences on causal relationships. Another limitation is self-reported data collection, such as the evaluation of NFL use or other health characteristics data, which can cause nondifferential misclassification bias and social desirability response bias. However, this method is commonly used and accepted in other survey studies (27,38–40). Additionally, the nutritional data provided in this survey relied solely on a single 24-hour recall, which may not accurately capture the full range of variation in participants’ habitual diets. Consequently, there is a possibility of both nondifferential information and regression dilution biases. Moreover, a selection bias exists due to the exclusion of patients with missing dietary data or invalid dietary recall, although the inclusion rate was high (87.2%). Finally, our study focused exclusively on adults aged 21 to 64 years, which limits the generalizability of our findings to people in other age groups.

In conclusion, by using NFLs, consumers can make more informed food choices that align with the principles of the DASH diet, such as selecting foods higher in protein, dietary fiber, and nutrients like calcium, magnesium, and potassium. Our study was conducted before the Israeli Ministry of Health’s new food labeling reform in January 2020 (41). This reform introduced a front-of-package system with mandatory red warning labels for sodium, saturated fat, and sugar, and an optional, positive (green) label to help consumers easily understand nutrition information and make healthier choices. Future studies will explore the reform’s effect on dietary habits and dietary patterns in Israel. We hope that this change will bring the population closer to DASH nutrient targets, particularly for sodium and saturated fat intake.
